# Distributed asynchronous measurement system fusion estimation based on inverse covariance intersection algorithm

**DOI:** 10.1038/s41598-024-54761-y

**Published:** 2024-02-17

**Authors:** Taishan Guo, Mingquan Wang, Shuyu Zhou, Wenai Song

**Affiliations:** 1 School of Instrumentation and Electronic, North University of China, Taiyuan, 030051 China; 2 Academy for Advanced Interdisciplinary Research, North University of China, Taiyuan, 030051 China; 3 School of Software, North University North University of China, Taiyuan, 030051 China

**Keywords:** Applied mathematics, Engineering, Mathematics and computing

## Abstract

For state estimation of multi-source asynchronous measurement systems with measurement missing phenomena, this paper proposes a distributed sequential inverse covariance intersection (DSICI) fusion algorithm based on conditional Kalman filtering method. It is mainly divided into synchronized state space module, local filtering module and fusion estimation module. The missing measurements occurring in the system are modelled and described by a set of random variables obeying a Bernoulli distribution. The synchronized state space module uses a state iteration method to synchronize the asynchronous measurement system at the moment of measurement update and it ensures the integrity of the measurement information. The local filtering module uses a conditional Kalman filtering algorithm for filter estimation. The reliability of the local filtering results is guaranteed because the local estimator designs a method to interact information with the domain sensors. The fusion estimation module designs a DSICI fusion algorithm with higher accuracy and satisfying consistency, which fuses the filtering results provided by each sensor when the relevant information between multiple sensors is unknown. Simulation examples demonstrate the excellent performance of the proposed algorithm, with a 33% improvement in accuracy over existing algorithms and an iteration time of less than 3 ms.

## Introduction

In the growing information age, single sensor systems can no longer satisfy the growing needs of people's life. In recent years, multi-sensor systems have been widely discussed as a popular research topic in the fields of airborne hierarchical networks^1^, power grid inspection^2^, disaster rescue^3^, intelligent logistics^4^, industrial control and address exploration^5,6^. This is due to the fact that multiple sensors can accomplish various kinds of complex tasks through collaborative sensing and information sharing, and have the advantages of low-cost and high-performance intelligent autonomy. These advantages are derived from the information fusion estimation theory. Therefore, information fusion estimation is an important research topic in multi-sensor information fusion technology^7–9^.

In multi-sensor systems, information fusion estimation methods are usually divided into centralized fusion estimation and distributed fusion estimation. The principle is to fuse multiple pieces of information into a more reliable one according to the corresponding fusion algorithm^10–12^. In centralized fusion systems, measurements from multiple sensors are processed using state measurement augmentation methods, which can yield excellent estimation results though. However, when one of the sensors fails or is damaged during operation, the centralized system cannot detect and discard the malfunctioning sensor in a timely manner, leading to a decrease in the reliability of the fusion estimation results and an increase in the error. In contrast, distributed fusion systems have a unique parallel structure. The existence of parallel structure makes it easy to detect and isolate faulty sensors and ensures the correctness of the fusion estimation results, so the distributed estimator has good reliability and flexibility^13^. In order to improve the reliability and flexibility of multi-sensor systems, it is important to use distributed fusion systems.

However, in a multi-sensor distributed fusion system, the sensors are transmitted to each other through a wireless network. Therefore, it is inevitable that the measurement data from sensors are delayed by the effects of network channel blockage and congestion. For example, the phenomenon of data transmission delay in UAVs, unmanned vehicles and unmanned ships cluster systems for high-precision positioning can reduce the credibility of the positioning results^14^. For the phenomenon of delayed measurement transmission, measurement enhancement techniques that combine current measurement with measurement delay and replication retransmission are often used to make full use of the delayed measurement data^15^. However, the measurement enhancement technique solves the fusion estimation problem through the augmentation technique, which inevitably results in an increase in computational complexity. Literature^16^ utilizes a Variational Bayes (VB) based approach. It combines augmented state vectors, random variables and covariance matrices for joint estimation, which effectively solves the occurrence of one-step random delayed measurement and measurement noise phenomena^17,18^.

Meanwhile, the occurrence of delay phenomenon must be accompanied by packet loss, which greatly affects the performance of the system. In order to ensure the integrity of the measurement data, literature^15^ investigates the optimal filtering problem of the random missing system with Markov chain communication measurement, which effectively solves the description of the missing measurement phenomenon. For the uncertain measurement missing phenomenon, an adaptive Kalman filter based on VB was used in literature^19^ to solve the filtering problem of unknown measurement missing. Its method is more accurate compared to existing filtering methods. Meanwhile, a set of Bernoulli distributed random variables is proposed in the literature^20^ to describe the random measurement missing phenomenon, and good results are obtained. Although, the measurement delay phenomenon can be achieved by increasing the sampling rate of the hardware system, but this will greatly increase the cost of using the system. Therefore, how to deal with the measurement delay phenomenon in a low-cost and effective way is a very urgent problem.

In daily life, for multi-sensor systems, the measurement sampling frequency varies between sensors because of the inconsistent operating performance of the sensors. In the sensor measurement sampling work, when the sensor measurement sampling frequency is different from the state update frequency, the system will have the problem of asynchronization of the sensor measurement update period and the state update period^21^. This phenomenon will lead to errors in the fusion results if the sensor sampling is not synchronized. In general, since the sensor sampling frequency is always smaller than the state update frequency, a commonly adopted method is to use state iteration^22^. It involves transforming the original system with asynchronous sampling into a state space model that is sampled synchronously at the moment of measurement sampling^23^. However, the sampling moment of the synchronized state space model is only at the sensor sampling moment, so it leads to the missing information of the state update point of the system, which seriously affects the filter's true estimation of the state information^24,25^. Therefore, how to effectively synchronize the multi-sensor asynchronous measurement system is one of the focuses of this paper.

For distributed information fusion theory, distributed fusion technique ensures the reliability and flexibility of the system. The fusion technique is processed by the correlation information between sensors. However, in multi-sensor systems, as the correlation information between sensors is difficult to be acquired, it leads to the inability to fuse the filtering results provided by each sensor^26^. Therefore, in this paper, the unknown correlation required for multi-sensor fusion is analyzed. Currently, the main methods that can solve the fusion estimation with unknown correlation are: Covariance Intersection (CI) fusion method, Ellipsoidal Intersection (EI) fusion method, and Inverse Covariance Intersection fusion method (ICI). CI fusion method is achieved by parameterizing the fusion formula, and it bypasses the fusion formula. by parameterizing the fusion formula, which bypasses the discussion of correlation information between sensors and describes it by finding a minimizing ellipsoid that characterizes the common information between sensors^27^. While this approach is easy to understand, the lack of discussion of unknown correlations inevitably leads to overly conservative fusion results^28^. In the pursuit of estimation results with higher accuracy, an EI fusion approach is proposed in the literature^29^ to redefine a parametric method. It ensures the accuracy of the fusion estimation results by finding the maximization ellipsoid that can characterize the correlation information and describing out the unknown correlation information with explicit expressions^30^.

However, EI fusion techniques cannot fully guarantee the consistency problem, which leads to the fact that EI fusion can only be adapted to some specific systems. In order to obtain the consistency that satisfies the fusion results and improve the fusion accuracy at the same time. Literature^31^ proposes an ICI fusion method, which starts from the perspective of the inverse of the covariance matrix and finds the inverse of the correlation information by the boundary range information of the inverse covariance matrix, while describing the correlation information to ensure the consistency problem of the fusion results^32^.

**Figure 1:**
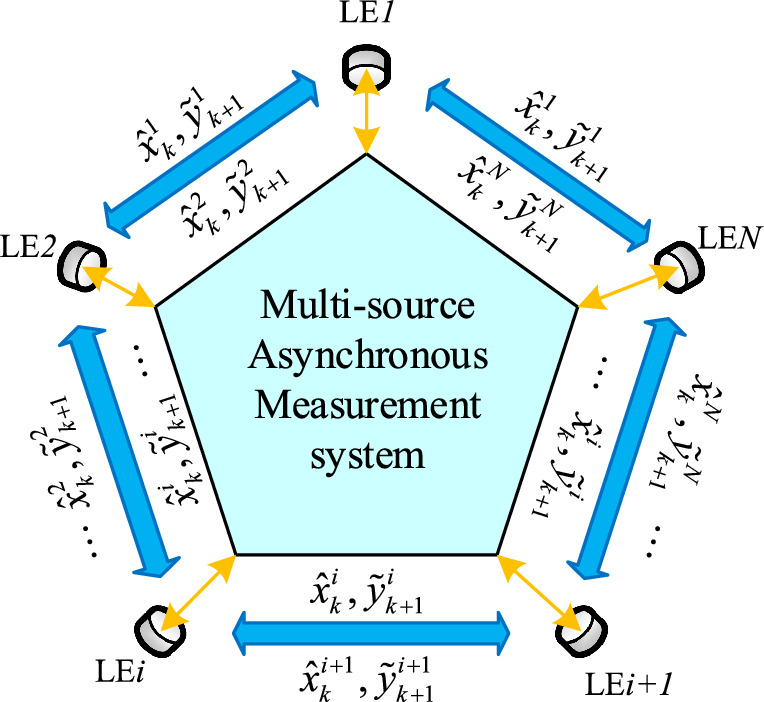
General system block diagram.

In summary, in order to solve the problems of asynchronous measurements, measurement delays and unknown correlations occurring in multi-sensor systems, so as to design a low-cost and high-precision distributed fusion algorithm to estimate the system. Therefore, in this paper, for the state estimation problem of multi-source asynchronous measurement system with measurement missing phenomenon induced by network channel blocking, a Distributed Sequential Inverse Covariance Intersection (DSICI) fusion estimator based on conditional Kalman filtering algorithm is proposed. The system is mainly divided into a synchronized state space module, a local filtering module and a fusion estimation module. The system block diagram is shown in Figure 1.

For asynchronous measurements occurring in the system, the Synchronized State Space module uses a state iteration method to transform the multi-source asynchronous measurement system into a state space model that is sampled synchronously at the moment of measurement update. It ensures the complete filtering effect. Due to the occurrence of measurement missing phenomenon induced by network channel blockage or congestion in multi-sensor systems, this paper models and describes it by a set of random Bernoulli distributed variables. This approach ensures the completeness of the measurement information.

Secondly, for the design of the filter estimator, the local filter module is designed to filter the system by means of the conditional Kalman filtering algorithm. The filtered values are output at the moment of measurement update and the predicted values are output at the moment of state update. The local filter is also designed to interact information with the neighborhood information under the finite communication domain in order to ensure the information interaction between multiple sensors. It ensures the reliability of the local filter.

Finally, for the unknown correlation problem in the fusion process, the fusion estimation module selects the ICI fusion algorithm, which has a higher fusion estimation accuracy and meets the fusion consistency, to deal with the fusion estimation problem of the unknown correlation information between sensors. The distributed sequential approach ensures the reliability and flexibility of the fusion process of multi-source asynchronous measurement systems.

## Problem description

In the study of control theory, the linear model is deeply favored by researchers for its strict quantitative description of the system state information. However, in real life, considering that multiple sensors may work together in a dynamic control system, this phenomenon inevitably leads to the problem of mismatching measurement rates between sensors^15^. Therefore, for the realistic description and estimation of a multi-source dynamic control system, we consider a multi-source asynchronous measurement system whose system state space model is shown as follows:

1 $$ x\left( {k + 1} \right) = Ax\left( k \right) + D\omega \left( k \right) \, $$

2 $$ \begin{aligned} y_{i} \left( {g_{i} k} \right) & = H_{i} x\left( {g_{i} k} \right) + v_{i} \left( {g_{i} k} \right) \\ i \, & { = 1,2,} \cdots {,}N \\ \end{aligned} $$

where $x\left( k \right) \in R^{n}$ is the system state vector at time $k$.$y_{i} \left( {g_{i} k} \right) \in R^{{m_{i} }}$ denotes the measured value of the $i$ sensor at sampling moment $g_{i} k$, and $g_{i} k$ denotes the sampling period of the $i$ sensor. The process noise $\omega \left( k \right) \in R^{n}$ and measurement noise $v\left( {g_{i} k} \right) \in R^{{m_{i} }}$ obey uncorrelated random white noise with mean 0 and covariance matrix $Q_{\omega } > 0$ and $R_{i,k} > 0$, respectively. The coefficient matrices $A$, $D$ and $H$ are constant matrices with appropriate dimensions, respectively. The initial state $x\left( 0 \right) \in N\left( {\mu_{0} ,P_{0} } \right)$ is uncorrelated with $\omega \left( k \right)$ and $v\left( {g_{i} k} \right)$.

In multi-sensor systems, channel congestion occurs when multiple measurement messages are transmitted due to the limited bandwidth of the network channel. The channel congestion phenomenon induces the measurement delay problem, i.e., the link nodes fail to receive the measurement data at the update moment, resulting in packet loss. Since the measurement missing phenomenon occurs randomly, this paper de-scribes the phenomenon that triggers measurement missing by means of a set of random Bernoulli distribution variables^16^.

3 $$ \begin{aligned} y_{i} \left( {g_{i} k} \right) & = \gamma_{i} \left( {g_{i} k} \right)H_{i} x\left( {g_{i} k} \right) + v_{i} \left( {g_{i} k} \right) \\ \, i \, & { = 1,2,} \cdots {,}N \, \\ \end{aligned} $$

where $\gamma_{i} \left( {g_{i} k} \right)$ is a random process variable obeying Bernoulli distribution. When $\gamma_{i} \left( {g_{i} k} \right) = 1$, it means that the $i$ sensor successfully measures the system at moment $g_{i} k$. Conversely, the system can only get measurement noise.

In a multi-source asynchronous measurement system, since the measurement sampling period is a positive integer multiple of the state update period, it is necessary to convert the asynchronous sampling system into a state space model with synchronous sampling at the measurement sampling time. Taking a sensor with a measurement update period of $g_{i}$ as an example, the state equation with a state update period of $g_{i}$ can be obtained by performing $g_{i}$ iterations of Eq. (1).

4 $$ \begin{aligned} x\left( {g_{i} \left( {k + 1} \right)} \right) & = Ax\left( {g_{i} k + g_{i} - 1} \right) + D\omega \left( {g_{i} k + g_{i} - 1} \right) \\ & { = }A^{2} x\left( {g_{i} k + g_{i} - 2} \right) + AD\omega \left( {g_{i} k + g_{i} - 2} \right) \\ \, & { + }D\omega \left( {g_{i} k + g_{i} - 1} \right) \\ & = A^{{g_{i} }} x\left( {g_{i} k} \right) + A^{{g_{i} - 1}} D\omega \left( {g_{i} k} \right) + A^{{g_{i} - 2}} D\omega \left( {g_{i} k + 1} \right) \\ & + \cdots + D\omega \left( {g_{i} k + g_{i} - 1} \right) \, \\ \end{aligned} $$

Organize (4) to obtain:

5 $$ x\left( {g_{i} \left( {k + 1} \right)} \right) = A^{{g_{i} }} x\left( {g_{i} k} \right) + \sum\limits_{m = 1}^{{g_{i} }} {A^{m} D\omega \left( {g_{i} k + g_{i} - m} \right)} $$

In this way, the multi-source asynchronous sampling system (1) and (3) with measurement missing phenomenon is transformed into a multi-source synchronous sampling system. Its state space model is

6 $$ x\left( {g_{i} \left( {k + 1} \right)} \right) = A^{{g_{i} }} x\left( {g_{i} k} \right) + W\left( {g_{i} k} \right) $$

7 $$ \begin{aligned} y_{i} \left( {g_{i} k} \right) & = \gamma_{i} \left( {g_{i} k} \right)H_{i} x\left( {g_{i} k} \right) + v_{i} \left( {g_{i} k} \right) \\ \, i \, & { = 1,2,} \cdots {,}N \\ \end{aligned} $$

where $$W\left( {g_{i} k} \right) = \sum\limits_{m = 1}^{{g_{i} }} {A^{m} D\omega \left( {g_{i} k + g_{i} - m} \right)}$$.

Since the converted synchronous system only samples at moments that are positive integer multiples of the measurement update period, it is important to design a filter to estimate the state information at the complete moment in a reasonable way.

### Conditional Kalman-based interactive local filtering estimation method

For a multi-sensor system synchronized at the measurement sampling moments, in this section, a conditional Kalman local filter is designed to handle the system noise. First, the filtering results for the multi-source complex system (6)–(7) synchronized at the measurement sampling moment can be derived according to the Kalman filtering principle^20^. The predicted estimation results of the system are:

$$ \hat{x}_{i} \left( {g_{i} k|g_{i} \left( {k - 1} \right)} \right) = A^{{g_{i} }} \hat{x}_{i} \left( {g_{i} \left( {k - 1} \right)|g_{i} \left( {k - 1} \right)} \right) $$

The prediction error covariance matrix is:

8 $$ \begin{aligned} P_{i} \left( {g_{i} k|g_{i} \left( {k - 1} \right)} \right) & = E\left[ {e_{i}^{ - } \left( {e_{i}^{ - } } \right)^{T} } \right] \\ & { = }A^{{g_{i} }} P_{i} \left( {g_{i} \left( {k - 1} \right)|g_{i} \left( {k - 1} \right)} \right)\left( {A^{{g_{i} }} } \right)^{T} \, \\ \, & { + }Q_{W} \, \\ \end{aligned} $$

where $e_{i}^{ - }$ denotes the prediction error and $Q_{W}$ denotes the covariance matrix of the process noise of the synchronous system.

The filtering estimation results are expressed as:

9 $$ \begin{aligned} \hat{x}_{i} \left( {g_{i} k|g_{i} k} \right) = & A^{{g_{i} }} \hat{x}_{i} \left( {g_{i} \left( {k - 1} \right)|g_{i} \left( {k - 1} \right)} \right) \\ & + K_{i} \left( {g_{i} k} \right)\varepsilon_{i} \left( {g_{i} k} \right) \, \\ \end{aligned} $$

where $\varepsilon_{i} \left( {g_{i} k} \right)$ denotes the new interest of the filtering process, i.e., the observed value minus the predicted observed value.

10 $$ \begin{aligned} \varepsilon_{i} \left( {g_{i} k} \right) = & y_{i} \left( {g_{i} k} \right) - \gamma_{i} \left( {g_{i} k} \right)H_{i} \\ & \times A^{{g_{i} }} \hat{x}_{i} \left( {g_{i} \left( {k - 1} \right)|g_{i} \left( {k - 1} \right)} \right) \, \\ \end{aligned} $$

Due to the Kalman filtering gain, the variance $Q_{{\varepsilon_{i} }} \left( {g_{i} k} \right)$ of the new interest $\varepsilon_{i} \left( {g_{i} k} \right)$ is calculated as follows:

11 $$ Q_{{\varepsilon_{i} }} \left( {g_{i} k} \right) = H_{i} \left[ {A^{{g_{i} }} P_{{\tilde{x}}} \left( {g_{i} \left( {k - 1} \right)|g_{i} \left( {k - 1} \right)} \right)A^{{g_{i} T}} + DQ_{\omega } \left( {g_{i} \left( {k - 1} \right)} \right)D^{T} } \right]H_{i}^{T} + R\left( {g_{i} k} \right) \, $$

The gain matrix of the filter can be obtained by organizing Eq. (11).

12 $$ K_{i} \left( {g_{i} k} \right) = \left[ {A^{{g_{i} }} P_{{\tilde{x}}} \left( {g_{i} \left( {k - 1} \right)|g_{i} \left( {k - 1} \right)} \right)A^{{g_{i} T}} + DQ_{\omega } \left( {g_{i} \left( {k - 1} \right)} \right)D^{T} } \right]H_{i}^{T} \times Q_{{\varepsilon_{i} }}^{ - 1} \left( {g_{i} k} \right) $$

In turn, the error covariance matrix $P_{{\tilde{x}}} \left( {g_{i} k|g_{i} k} \right)$ of the filtered values can be obtained:

13 $$ P_{{\tilde{x}}} \left( {g_{i} k|g_{i} k} \right) = \left[ {I_{n} - K_{i} \left( {g_{i} k} \right)H_{i} } \right] \times \left[ {A^{{g_{i} }} P_{{\tilde{x}}} \left( {g_{i} \left( {k - 1} \right)|g_{i} \left( {k - 1} \right)} \right)A^{{g_{i} T}} + DQ_{W} \left( {g_{i} \left( {k - 1} \right)} \right)D^{T} } \right] $$

**Figure 2:**
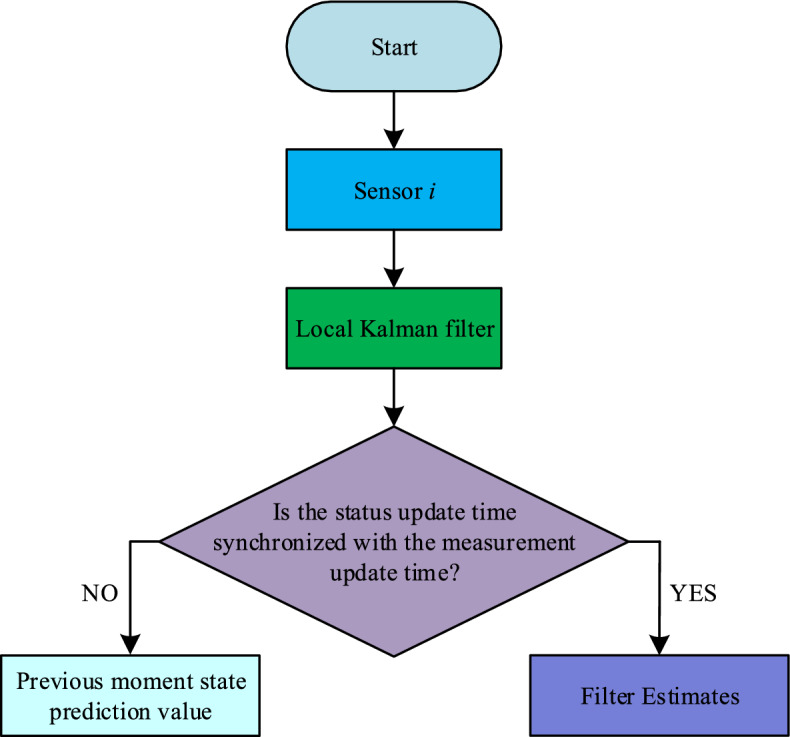
Conditional Kalman local filter flow chart.

Since the filtering results are only filtered at moments that are positive integer multiples of the measurement sampling period $g_{i}$, they do not provide a complete description of the state information. To solve this problem, the conditional Kalman filtering algorithm is used to design the state estimator^21^. The estimated amount $\hat{x}_{i,k}$ of state information at each moment and the estimation error covariance matrix $\hat{P}_{i,k}$ are discussed below in two cases. The flow chart of the conditional Kalman local filter is shown in Fig. 2.

Case 1: When the state update time is synchronized with the measurement sampling time, the state estimate is equal to the Kalman filter estimate at the measurement sampling time. The state estimation result $\left( {\hat{x}_{i,k} ,\hat{P}_{i,k} } \right)$ is:

14 $$ \hat{x}_{i,k} = \hat{x}_{i} \left( {g_{i} k|g_{i} k} \right){, }\hat{P}_{i,k} = P_{{\hat{x}}} \left( {g_{i} k|g_{i} k} \right) $$

Case 2: When the state update moment is not synchronized with the measurement sampling moment, the state estimate is described using the predicted value of the state estimate for the previous moment.

15 $$ \hat{x}_{i,k} = A\hat{x}_{i,k - 1} {, }\hat{P}_{i,k} = A\hat{P}_{i,k - 1} A^{T} + DQ_{\omega } D^{T} $$

At the same time, information interaction between multiple sensors can ensure the reliability of the multi-sensor system. In order to obtain effective local filter estimation results, we use the local filter as a node for information interaction between sensors, and the node carries out the interaction of the locally estimated filter values of other nodes in a limited communication domain. However, due to the limitation of the network channel capacity between sensors in a multi-sensor system, here we assume that the range of the effective communication domain of the local filter node communicates with only the two nearest nodes to itself^23^. Here, we take the $ith$ local filter node as an example, and the result of the information interaction in the limited communication domain is:

16 $$ \hat{x}_{i,k} = G_{i,k} \hat{x}_{i,k - 1} { + }L_{i,k} \sum\limits_{j \in N} {\left( {\hat{x}_{j,k - 1} - \hat{x}_{i,k - 1} } \right)} $$

17 $$ \hat{P}_{i,k} = G_{i,k} \hat{P}_{i,k - 1} { + }L_{i,k} \sum\limits_{j \in N} {\left( {\hat{P}_{j,k - 1} - \hat{P}_{i,k - 1} } \right)} \, $$

where $\hat{x}_{i,k}$ denotes the estimate at moment $k$ provided by the $ith$ local filter; $\hat{x}_{j,k - 1}$ denotes the state filter estimate provided by local filter $j$ in the effective communication domain; $G$ and $L$ denote the gain coefficients of each local filter estimation term in the state estimate. The gain coefficients are described by solving the optimization problem as follows:

18 $$ \mathop {\min }\limits_{{G_{i,k} ,L_{i,k} ,P_{i,k} \ge 0}} trace\left( {P_{i,k} } \right) \, $$

In a distributed system, it is crucial to select a suitable fusion method to fuse the filtering results of individual sensors in order to obtain more accurate estimation results.

## Distributed sequential inverse covariance intersection (DSICI) fusion estimator

In the multi-sensor data fusion process, data fusion algorithms provide more accurate fusion results by fusing in an efficient decision-making manner. However, for the fusion process of distributed systems, the fusion process is greatly troubled by the fact that the correlation information between sensors is not easily accessible. At present, for dealing with the fusion problem of unknown correlations, the commonly used fusion method is the CI fusion technique. The CI fusion technique defines the range of unknown correlations by finding a minimizing ellipsoid, and although this method is commonly accepted, the CI fusion technique focuses on analyzing the fusion formula rather than characterizing the correlations, so it can lead to overly conservative fusion results^28^. In order to obtain fusion estimation results with higher accuracy, the EI fusion technique explicitly describes the unknown correlation information by introducing a new estimator in terms of a representation formula, which precisely completes the fusion process by finding the ellipsoidal range of the maximized correlation information. And the EI fusion technique describes the optimization problem by an algebraic expression, which greatly reduces the computational complexity of the system^31^.

However, EI fusion techniques cannot fully guarantee the consistency problem. EI fusion techniques can guarantee consistent estimation only under certain specific conditions. This leads to the fact that EI fusion can only be adapted to some specific systems. Literature^32^ proposes an inverse covariance intersection (ICI) fusion method, which starts from the perspective of the inverse of the covariance matrix and finds the inverse of the correlation information by the boundary range information of the inverse covariance matrix, while describing the correlation information to ensure the consistency problem of the fusion results^33^. In addition, compared with the CI fusion results, ICI fusion can provide more rigorous fusion results and overcome the conservativeness problem of CI fusion results. This method avoids both conservative estimation and satisfies the consistency problem of the fusion results, and the accuracy of the fusion results is greatly guaranteed.

In the process of multi-sensor data fusion, the general equation for two-sensor based fusion estimation is as follows:

19 $$ \hat{x}_{fus} { = }K_{fus} \hat{x}_{A} { + }L_{fus} \hat{x}_{B} \, $$

where $K_{fus}$ and $L_{fus}$ denote the fusion gain and the corresponding covariance matrix is expressed as:

20 $$ P_{fus} { = }K_{fus} P_{A} K_{fus}^{T} { + }K_{fus} P_{AB} L_{fus}^{T} { + }L_{fus} P_{BA} K_{fus}^{T} { + }L_{fus} P_{B} L_{fus}^{T} \, $$

where $P_{AB} = P_{BA}$ denotes the cross-covariance matrix, and the fusion gain is determined by solving the trace of the minimized covariance matrix. So, the key to solve the fusion problem is to obtain the information of the cross-covariance matrix between sensors.

However, the acquisition of cross-covariance matrix information is very difficult in multi-source systems. So, this reason causes the correlation between multiple sensors to become unknown. Therefore, we analyze the existing fusion methods (CI fusion, EI fusion and ICI fusion techniques) for which the correlation is unknown. The first fusion method to emerge is CI fusion, which is widely used by virtue of achieving consistency in fusion results. The CI fusion process is shown below:

$$ \hat{x}_{fus}^{CI} = P_{fus}^{CI} \left[ {\omega P_{A}^{ - 1} \hat{x}_{A} + \left( {1 - \omega } \right)P_{B}^{ - 1} \hat{x}_{B} } \right] $$

21 $$ P_{fus}^{CI} = \left[ {\omega P_{i}^{ - 1} + \left( {1 - \omega } \right)P_{j}^{ - 1} } \right]^{ - 1} $$

where the optimal weight value $\omega$ satisfies:

22 $$ \mathop {\min }\limits_{0 \le \omega \le 1} trace\left( {P_{fus}^{CI} } \right) \, $$

Since the CI fusion process directly parameterizes the fusion Eq. (19), but it lacks the description of the cross-covariance matrix information, making the CI fusion method then needs to provide a large range of fusion results. Although CI fusion can guarantee the consistency of fusion results, the fusion results are too conservative.

The EI fusion method proposes to solve the problem of overly conservative fusion results. It describes the unknown correlation information explicitly by finding an ellipsoid that maximizes the information containing the cross-covariance matrix. Explicit expressions can also reduce the computational cost of the fusion process. The EI fusion process is described as follows:

$$ \hat{x}_{fus}^{EI} = P_{fus}^{EI} \left( {P_{A}^{ - 1} \hat{x}_{A} + P_{B}^{ - 1} \hat{x}_{B} - \Gamma^{ - 1} \gamma } \right) $$

23 $$ P_{fus}^{EI} = \left( {P_{A}^{ - 1} + P_{B}^{ - 1} - \Gamma^{ - 1} } \right)^{ - 1} $$

where the correlation means $\gamma$ and variances $\Gamma$ are expressed through the $x_{A}$ and $x_{B}$ information.

$$ \Gamma = S_{A} \sqrt {D_{A} } S_{B} D_{\Gamma } S_{B}^{ - 1} \sqrt {D_{A} } S_{A}^{ - 1} $$

24 $$ \begin{aligned} \gamma = & \left( {P_{A}^{ - 1} + P_{B}^{ - 1} - 2\Gamma^{ - 1} + 2\eta I} \right)^{ - 1} \\ & \times \left( \begin{gathered} \left( {P_{B}^{ - 1} - \Gamma^{ - 1} + \eta I} \right)\hat{x}_{A} \hfill \\ + \left( {P_{A}^{ - 1} - \Gamma^{ - 1} + \eta I} \right)\hat{x}_{B} \hfill \\ \end{gathered} \right) \, \\ \end{aligned} $$

where $S$ denotes the feature vector matrix and $D$ denotes the feature diagonal matrix.

However, the EI fusion method can guarantee the consistency of fusion results only under some specific conditions. In order to obtain a fusion method with high fusion accuracy and satisfy the consistency of fusion results, ICI fusion is proposed^34^. It starts from the perspective of the inverse of the covariance matrix and describes the unknown correlation information by finding the boundary information of the intersection region of the inverse covariance matrix. The process of ICI fusion is as follows:

$$ \hat{x}_{fus}^{ICI} = W_{A} \hat{x}_{A} + W_{B} \hat{x}_{B} $$

25 $$ P_{fus}^{ICI} = \left( {P_{A}^{ - 1} + P_{B}^{ - 1} - \left( {\omega P_{A} + \left( {1 - \omega } \right)P_{B} } \right)^{ - 1} } \right)^{ - 1} $$

where the fusion weights $W_{A}$ and $W_{B}$ satisfy the following rules.

$$ W_{A} = P_{fus}^{ICI} \left( {P_{A}^{ - 1} - \omega \left( {\omega P_{A} + \left( {1 - \omega } \right)P_{B} } \right)^{ - 1} } \right) $$

26 $$ W_{B} = P_{fus}^{ICI} \left( {P_{B}^{ - 1} - \left( {1 - \omega } \right)\left( {\omega P_{A} + \left( {1 - \omega } \right)P_{B} } \right)^{ - 1} } \right) $$

The optimal value of the parameter $\omega$ is determined by $\mathop {\min }\limits_{\omega } trace\left( {P_{fus}^{ICI} } \right)$.

**Figure 3:**
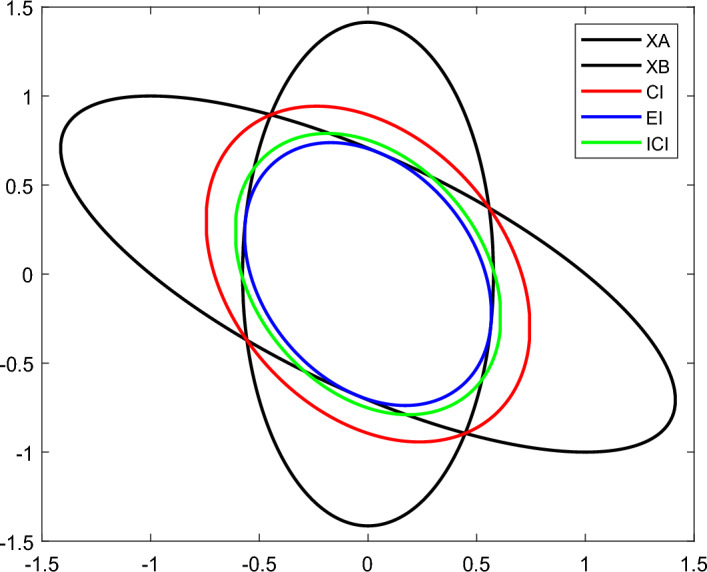
Comparison of the fusion results of CI, EI and ICI fusion algorithms.

Next, we compare the differences of the three fusion methods by a numerical example. Suppose two random variables $X_{A}$ and $X_{B}$ obeying Gaussian distribution^35^. $X_{A}$ obeys a mean value of $$x_{A} = \left[ {\begin{array}{*{20}c} 0 & 0 \\ \end{array} } \right]^{T}$$ and a covariance matrix of $P_{A} = \left[ {2, - 1; - 1,1} \right]$; $X_{B}$ obeys a mean value of $$x_{B} = \left[ {\begin{array}{*{20}c} 0 & 0 \\ \end{array} } \right]^{T}$$ and a covariance matrix of $P_{B} = \left[ {1/3,0;0,2} \right]$. The fusion results of the three fusion methods are shown in Fig. 3.

The area enclosed by the red curve indicates the fusion result of the CI fusion method, the area enclosed by the blue curve indicates the fusion result of the EI fusion method, and the area enclosed by the green curve indicates the fusion result of the ICI fusion method. The results show that the area enclosed by the CI fusion algorithm is the largest. It over-describes the public information of the two random variables, which leads to the over-conservative CI fusion estimation results. the EI fusion estimation results guarantee the accuracy of the fusion results. However, the consistency problem of the EI fusion results is not clearly demonstrated at present.

Based on the above analysis, we select ICI fusion technique for the design of distributed fusion estimator. Also, to reduce the computational cost of the multi-sensor system, we design the fusion process as sequential fusion by the fusion process requires $N - 1$ times of fusion of two-two local filter estimates. The distributed sequential inverse covariance intersection (DSICI) fusion estimation process is shown as follows:

$$ x_{s,k}^{0} = \hat{x}_{1,k} ,P_{s,k}^{0} = \hat{P}_{1,k} $$

$$x_{s,k}^{i} = W_{a}^{i} x_{s,k}^{i - 1} + W_{b}^{i} \hat{x}_{i,k}$$

27 $$ P_{s,k}^{i} = \left( {\left( {P_{s,k}^{i - 1} } \right)^{ - 1} + \hat{P}_{i + 1,k}^{ - 1} - \left( {\omega_{i} P_{s,k}^{i - 1} + \left( {1 - \omega_{i} } \right)\hat{P}_{i + 1,k} } \right)^{ - 1} } \right)^{ - 1} $$

where the expressions for $W_{a}^{i}$ and $W_{b}^{i}$ are:

$$ W_{a}^{i} = P_{s,k}^{i} \left( {\left( {P_{s,k}^{i - 1} } \right)^{ - 1} - \omega_{i} \left( {\omega_{i} P_{s,k}^{i - 1} + \left( {1 - \omega_{i} } \right)\hat{P}_{i + 1,k} } \right)^{ - 1} } \right) $$

$$ W_{b}^{i} = P_{s,k}^{i} \left( {\hat{P}_{i + 1,k}^{ - 1} - \left( {1 - \omega_{i} } \right)\left( {\omega_{i} P_{s,k}^{i - 1} + \left( {1 - \omega_{i} } \right)\hat{P}_{i + 1,k} } \right)^{ - 1} } \right) $$

28 $$ \mathop {\min }\limits_{{0 \le \omega_{i} \le 1,\sum {\omega_{i} } = 1}} trace\left( {P_{s,k}^{i} } \right) $$

At the same time, we analyzed the consistency of the fusion results of the DSICI fusion estimator. First, a comparison of the traces of the error covariance matrix of the first fusion results is shown below:

29 $$ tr\left( {P_{s,k}^{1} } \right) \le tr\left( {P_{s,k}^{0} } \right), \, tr\left( {P_{s,k}^{1} } \right) \le tr\left( {\hat{P}_{2,k} } \right) $$

Then, the results of the second fusion can be obtained based on the iterative approach.

$$ tr\left( {P_{s,k}^{2} } \right) \le tr\left( {P_{s,k}^{1} } \right), \, tr\left( {P_{s,k}^{2} } \right) \le tr\left( {\hat{P}_{3,k} } \right) $$

The collation gives:

30 $$ tr\left( {P_{s,k}^{2} } \right) \le tr\left( {\hat{P}_{i,k} } \right),i = 1,2,3 $$

Based on the mathematical induction method, the final fusion error covariance matrix is smaller than the error covariance matrix provided by each filter in the DSICI fusion estimator, which undergoes $N - 1$ times of EI fusion process.

31 $$ tr\left( {P_{s,k}^{N - 1} } \right) \le tr\left( {P_{i,k} } \right),i = 1,2,3, \cdots ,N \, $$

Through the above analysis, the DSICI fusion estimator designed in this paper has good consistency and the fusion estimator outperforms the individual filter estimators.

## Simulation experiment analysis

In order to visually verify the proposed DSEI fusion estimation algorithm based on conditional Kalman filtering, the reliability of the estimation results of a multi-source asynchronous measurement system with measurement missing phenomena is guaranteed. In this section, to ensure the boundedness of the system state values, the superior performance of the DSICI fusion estimator is verified by constructing a stable time-varying linear numerical example. The multi-source asynchronous sampling system with measurement deficiencies is shown as follows:

$$ x\left( {k + 1} \right) = Ax\left( k \right) + D\omega \left( k \right) $$

$$ \begin{gathered} y_{i} \left( {g_{i} k} \right) = \gamma_{i} \left( {g_{i} k} \right)H_{i} x\left( {g_{i} k} \right) + v_{i} \left( {g_{i} k} \right) \\ \, i{ = 1,2,}3 \, \\ \end{gathered} $$

where the state variables $x\left( k \right) = \left[ {x_{1} \left( k \right),x_{2} \left( k \right),x_{3} \left( k \right)} \right]^{T}$, The measurement periods of the measurement equation are $g_{1} = 1$, $g_{2} = 2$ and $g_{3} = 3$. The state coefficient matrix $A = diag\left( {a_{1} ,a_{2} ,a_{3} } \right)$, the expressions of the elements of each matrix are:

$$ a_{1} = \exp \left[ { - h + \sin \left( h \right)} \right] $$

$$a_{2} = 2\sinh \left( {h/2} \right)\exp \left[ { - h + \sin \left( h \right)} \right]$$

$$ a_{3} = \exp \left[ { - 2h + \sin \left( h \right)} \right] $$

The measurement matrix of each sensor is:

$$ H_{1} = \left[ {1,\cos \left( h \right),\sin \left( h \right)} \right] $$

$$ H_{2} = \left[ {\sin \left( h \right),2,\cos \left( h \right)} \right] $$

$$ H_{3} = \left[ {\cos \left( h \right),\sin \left( h \right),1.5;1,\sin \left( {2h} \right),\cos \left( {2h} \right)} \right] $$

The System parameters is $h = 0.2$. The measurement misses occurring in each sensor in the system are described by random variables $\gamma$ that obey a Bernoulli distribution, with variable parameters $\gamma_{1} = 0.9$, $\gamma_{2} = 0.7$, $\gamma_{3} = 0.4$. The coefficient matrix $D$ and covariance matrix of the process noise are $Q = diag\left( {1,1,1} \right)$ , and the covariance matrices of the measurement noise are $R_{1} = 0.2$, $R_{2} = 0.3$ and $R_{3} = \left[ {0.3,0.1;0.1,0.25} \right]$, respectively. The initial state values and covariance matrices are:

$$ x_{0} = \left[ {0.1;0.1;0.1} \right],\;\;P_{0} = diag\left( {0.1,0.1,0.1} \right) $$

In order to accurately evaluate the performance of the proposed DSICI fusion estimation algorithm, the reliability of the algorithm is verified by analyzing the Root Mean-Square Error (RMSE) of the estimation results.

$$ \begin{gathered} RMSE_{i,k}^{l} = \sqrt {\frac{1}{N}\sum\limits_{n = 1}^{N} {\left[ {x_{k}^{l} \left( n \right) - \hat{x}_{i,k}^{l} \left( n \right)} \right]^{2} } } \hfill \\ \, i = 1,2,3;l = 1,2,3 \hfill \\ \end{gathered} $$

where $\hat{x}_{i,k}^{l}$ denotes the $ist$ component of the $1st$ conditional local filter value and $N = 5000$ is the number of Monte Carlo runs.

**Figure 4:**
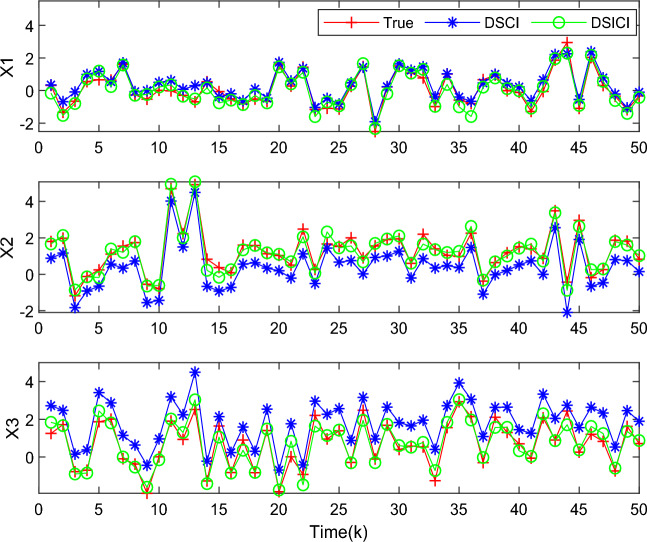
State tracking performance of DSCI and DSICI fusion estimation algorithms.

The proposed DSICI fusion estimation algorithm based on conditional Kalman filtering is compared with the existing Distributed Sequential Covariance Intersection (DSCI) fusion estimation algorithm by conducting simulation experiments to obtain the estimation results of the state information. The state tracking performance results are shown in Fig. 4. The red curve shows the actual state value of the multi-source asynchronous measurement system with measurement delay phenomenon, the blue curve shows the DSCI fusion estimation, and the green curve shows the DSICI fusion estimation. It can be seen that DSICI achieves better tracking performance for the state variables compared to the fusion estimation results of DSCI.

**Figure 5:**
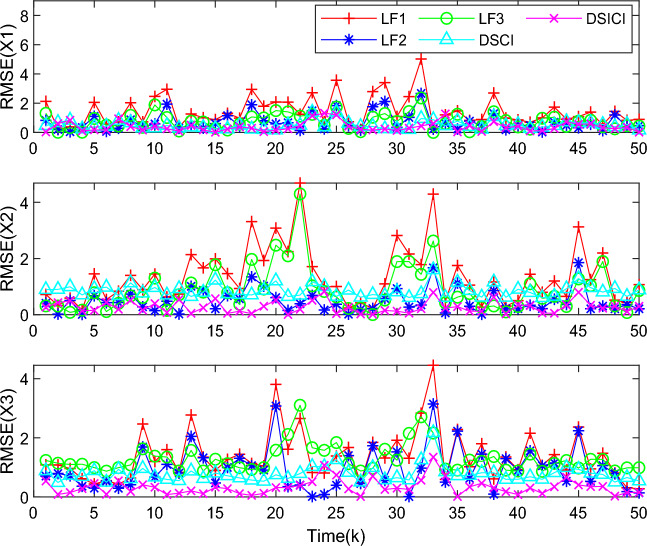
Comparison of RMSE of local filtering with DSCI and DSICI fusion estimation results.

Meanwhile, to further validate the performance of the DSICI fusion estimator. In this paper, by analyzing the RMSE of the estimation results of the local filter estimator, DSCI and DSICI fusion estimator, the comparison of the RMSE results of each local filter estimator, DSCI and DSICI fusion estimator is shown in Fig. 5. The results show that the RMSEs of each state of the DSICI fusion estimation results are smaller than those of the local filter estimation. Meanwhile, the RMSE of the DSICI fusion estimation results is also smaller than that of the DSCI fusion results, which ensures the accuracy of the fusion results.

**Figure 6:**
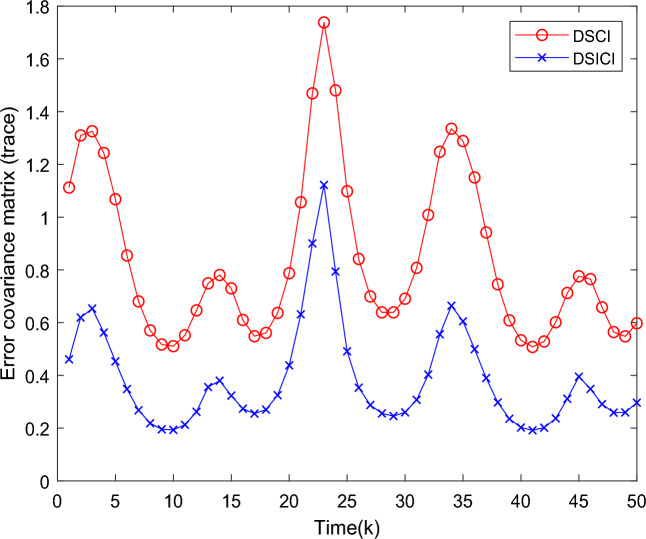
Comparison of the traces of the error covariance matrix of the DSCI and DSICI fusion estimation.

Further, to verify the good estimation performance of DSICI fusion algorithm compared to DSCI fusion algorithm in distributed fusion system. In this paper, we compare the traces of the error covariance matrix of DSCI and DSICI fusion estimation, i.e., the smaller the trace of the matrix, the smaller the error range. The comparison of the traces of the error covariance matrix of the two methods is shown in Fig. 6. The results show that the traces of the error covariance matrix of the DSCI fusion results are larger than those of the DSICI fusion, and the error range of DSICI is reduced to 33% of that of DSCI, which greatly improves the reliability of the system fusion results.

**Figure 7:**
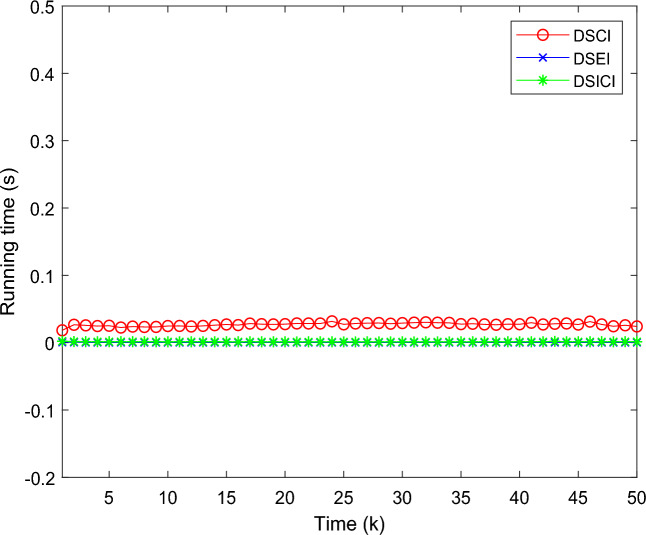
Comparison of DSCI, DSEI and DSEI fusion estimated running times.

Also, this paper compares the computational time cost of DSCI fusion, DSEI fusion and DSICI fusion methods in the running process, and the comparison results are shown in Fig. 7. The results show that the iterative running time of the DSICI fusion estimation method is smaller than that of the DSCI fusion estimation method. Among them, the iterative running time of DSICI fusion estimation method is about 0.002885s, which can be controlled within the range of 3ms. While the iterative running time of DSCI is about 0.030756s. The iterative running times of the three fusion methods are shown in Table 1. The reason for the low computational cost of the DSEI fusion algorithm is that the fusion process optimization problem is described explicitly, and the explicit expression will greatly reduce the computational cost reduction of the running process, thus accelerating the output of the fusion results.

**Table 1:** The iterative running times of the DSCI, DSEI and DSICI fusion method.

Fusion method	DSCI	DSEI	DSICI
Running time	0.030756 s	0.002798 s	0.002885 s

## Conclusion

In this paper, we address the aspects of asynchronous sampling, measurement delays and unknown correlations that occur in multi-sensor systems. We propose a DSICI fusion estimation algorithm based on the conditional Kalman filtering method. For the phenomenon of measurement delay that occurs in multi-source asynchronous measurement systems, a set of random variables obeying the Bernoulli distribution is used to describe it, and, in this paper, we use the state iteration method to synchronize the multi-source asynchronous measurement systems at the moment of measurement update. It ensures the completeness and accuracy of the measurement data. For the problem of state noise and measurement noise in the system, the local filter adopts the conditional Kalman filtering algorithm to filter the system noise, and outputs the filtered value when the state update is synchronized with the measurement update moment, otherwise outputs the state prediction value, which is a complete filtered estimation of the state information. For the problem of unknown correlation between sensors, this paper adopts the DSICI fusion algorithm to fuse the filtering results provided by local filters. The fusion estimation error accuracy of this method is improved by 33% compared with DSCI, and the iterative running time can be controlled within 3ms, which ensures the reliability of the fusion results and reduces the computational cost of the system. Although, the algorithm proposed in this paper is feasible after simulation data analysis. However, the implementation of this paper's algorithm transplanted to the hardware platform (Unmanned vehicles, UAV cluster cooperative positioning operations and other fields) is worthy of the author's deep thought.

## Data Availability

The datasets used and/or analyzed during the current study available from the corresponding author on reasonable request.
